# External Validation and Extension of a Cochlear Implant Performance Prediction Model: Analysis of the Oldenburg Cohort

**DOI:** 10.3390/audiolres15030069

**Published:** 2025-06-12

**Authors:** Rieke Ollermann, Robert Böscke, John Neidhardt, Andreas Radeloff

**Affiliations:** 1 Human Genetics, Faculty of Medicine and Health Science, University of Oldenburg, 26129 Oldenburg, Germany; 2 Division of Otolaryngology, Head and Neck Surgery, University of Oldenburg, 26129 Oldenburg, Germany; 3 Research Center Neurosensory Science, University of Oldenburg, 26129 Oldenburg, Germany; 4 Cluster of Excellence “Hearing4All”, University of Oldenburg, 26129 Oldenburg, Germany

**Keywords:** prediction of CI outcome, Freiburg monosyllabic speech test, severe-to-profound hearing loss, CI manufacturer, bilateral hearing loss

## Abstract

**Background/Objectives**: Rehabilitation success with a cochlear implant (CI) varies considerably and identifying predictive factors for the reliable prediction of speech understanding with CI remains a challenge. Hoppe and colleagues have recently described a predictive model, which was specifically based on Cochlear™ recipients with a four-frequency pure tone average (4FPTA) ≤ 80 dB HL. The aim of this retrospective study is to test the applicability to an independent patient cohort with extended inclusion criteria. **Methods**: The Hoppe et al. model was applied to CI recipients with varying degrees of hearing loss. Model performance was analyzed for Cochlear™ recipients with 4FPTA ≤ 80 dB HL and for all recipients regardless of 4FPTA. Subgroup analyses were conducted by *WRS_max_* and CI manufacturer. **Results**: The model yielded comparable results in our patient cohort when the original inclusion criteria were met (n = 24). Extending the model to patients with profound hearing loss (4FPTA > 80 dB HL; n = 238) resulted in a weaker but significant correlation (r = 0.273; *p* < 0.0001) between predicted and measured word recognition score at 65 dB with CI (*WRS*_65_(*CI*)). Also, a higher percentage of data points deviated by more than 20 pp, either better or worse. When patients provided with CIs from different manufacturers were enrolled, the prediction error was also higher than in the original cohort. In Cochlear™ recipients with a maximum word recognition score (*WRS_max_*) > 0% (n = 83), we found a moderate correlation between measured and predicted scores (r = 0.3274; *p* = 0.0025). **Conclusions**: In conclusion, as long as the same inclusion criteria are used, the Hoppe et al. (2021) prediction model results in similar prediction success in our cohort, and thus seems applicable independently of the cohort used. Nevertheless, it has limitations when applied to a broader and more diverse patient cohort. Our data suggest that the model would benefit from adaptations for broader clinical use, as the model lacks sufficient sensitivity in identifying poor performers.

## 1. Introduction

Cochlear implant (CI) provision is the standard treatment for patients with severe to profound hearing loss. Over the last decades, the indication criteria have changed considerably. Current guidelines in Germany recommend cochlear implantation “for patients who are likely to achieve better hearing and speech understanding with cochlear implants than with conventional hearing aids or other implantable hearing systems” [1] (p. 7). The majority of patients benefit significantly from CI provision. However, the success of rehabilitation is still highly variable and there is a small risk that speech understanding may be worse after surgery than before. A reliable individual prediction of the postoperative CI performance would therefore be helpful.

Several models have been proposed either to identify predictive factors for CI outcomes or to predict CI outcomes, especially in terms of speech understanding. Of these models, some used correlation analyses to identify predictors for outcome based, for example, on multiple regression analyses [2,3,4,5,6,7]. Other models predicted the CI outcome by using general linear model analysis [8,9,10,11] and machine learning models [12,13]. These models included several different predictive factors such as age at onset of hearing loss (HL), duration of HL, age at implantation, preoperative speech recognition, preoperative pure tone average (PTA) and underlying etiology to name a few [11,13]. Different studies revealed different predictors. Blamey et al. [2,3] showed that age at onset of HL, duration of HL, age at implantation, underlying etiology and duration of CI experience have different effects on the outcome [3]. Lazard et al. [14] extended the model proposed by Blamey et al. [2,3] by adding factors such as the surgical approach, the insertion angle, hearing aid use and duration of the moderate hearing loss. PTA of the better hearing ear, hearing aid use, CI manufacturer, percentage of active electrodes and duration of a moderate hearing loss were found to be significant predictors of CI outcome. In another study, four different machine learning models (artificial neural network, random forest and gradient boosting) were applied based on demographic factors, audiological and hearing-related metrics, patient history and etiology [13]. The performance of these models was compared with the more traditional generalized linear model (GLM) under conditions used in Blamey et al. [3] and Lazard et al. [14] and the reported random forest regression model from Kim et al. [12] to assess machine learning model accuracy and generalizability. While known variables provide some insight, they only explain 10–60% of variability in CI performance [2,3,7,14]. In summary, it remains a challenge to develop a reliable predictive model for cochlear implant outcomes. A major obstacle is the dependence of many models on factors that can only be collected anamnestically, which introduces uncertainty.

Hoppe and colleagues [11] developed a GLM for postoperative word recognition scores with CI based on the following objective factors that can be measured during preoperative assessment: “age at implantation”, “maximum word recognition score” and “word recognition score with hearing aids”. They selectively included patients who met the following criteria: (i) preoperative four-frequency pure tone average (4FPTA) ≤ 80 dB HL, (ii) hearing aid use for at least 3 months before surgery, (iii) participation in CI rehabilitation for at least 6 months after surgery, and (iv) implanted CIs from the manufacturer Cochlear Deutschland GmbH & Co. KG, Hannover, Germany.

The aim of our study is to examine the applicability of the prediction model developed by Hoppe et al. [11] to an independent cohort and to a broader set of inclusion criteria, encompassing additional CI manufacturers.

## 2. Patients and Methods

This study was approved by the local authorities responsible (Medical Ethics Committee application #2024-021). We retrospectively collected data from 803 adult patients who received a CI at our tertiary referral center. Patients underwent implantation between 2007 and 2022. Patients with etiologies that adversely affect CI performance, such as multiple sclerosis, auditory synaptopathy and neuropathy and vestibular schwannoma were excluded [3]. Additional exclusion criteria were factors such as prelingual or perilingual deafness, non-German first language, intellectual disabilities and absence of prior hearing aid use. After applying these criteria, 238 eligible patients remained for analysis.

Of these 238 patients, we analyzed several subcohorts. First, we identified 24 subjects matching the following inclusion criteria established by Hoppe et al. [11] (with one modification—our patients underwent at least 12 months of CI rehabilitation):

- Preoperative 4FPTA ≤ 80 dB HL.
- Native language: German.
- Sensory neural hearing loss.
- At least 6 months of CI rehabilitation.
- Experience of hearing with hearing aids.
- Manufacturer: Cochlear.

Second, we expanded this subcohort to include patients with implants from other manufacturers (MED-EL and Advanced Bionics) while maintaining all other criteria, resulting in 51 patients.

Finally, in order to comprehensively address our study objectives, we analyzed the entire cohort of 238 CI recipients, regardless of 4FPTA value and manufacturer, while maintaining all other inclusion criteria.

### 2.1. Audiometry

We derived the 4FPTA from hearing level thresholds at 0.5–4 kHz obtained from pure tone audiograms. From the Freiburg monosyllabic word test, we extracted the following three key measurements: the preoperative unaided maximum word recognition score (*WRS_max_*), the preoperative aided word recognition score with hearing aids (*WRS*_65_(*HA*)) and the postoperative word recognition score with CI (*WRS*_65_(*CI*)).

Measurement of the *WRS*_65_(*HA*) was performed in a free field with loudspeakers placed at a distance of 1 m and an azimuth of 45° to the patient’s defective ear. The contralateral ear was masked with white noise according to Comité Consultatif International Téléphonique et Télégraphique (CCITT; [15]) via headphones.

Postoperatively, the *WRS*_65_(*CI*) was measured at the 1-year postoperative follow-up. The setup for this measurement was the same as for the *WRS*_65_(*HA*).

### 2.2. Prediction Model

Hoppe et al. [11] developed the following model to predict the performance with CI using a generalized linear model (GLM) for logistic regression:

(1) $$WRS_{65}\left(CI\right)\left[\%\right]=\frac{100}{1+e^{-\left(\beta_{0}+\beta_{1}\cdot WRS_{max}+\beta_{2}\cdot age+\beta_{3}\cdot WRS_{65}\left(HA\right)\right)}}$$

The estimates of the model parameters are provided in Table 1.

This equation linearly combines the variables *WRS_max_*, age at implantation and *WRS*_65_(*HA*) to compute a prediction and applies a logistic function to convert the predicted outcome to a percentage. The coefficient *β*_0_ represents the intercept term, which establishes the baseline value when all predictor variables equal zero. *β*_1_, *β*_2_ and *β*_3_ are the coefficients representing how strongly each predictor influences the outcome. For example, a positive *β*_1_ and/or *β*_3_ indicates that better preoperative performance in either *WRS_max_* or hearing aid performance improves the likelihood of higher *WRS*_65_(*CI*) [%]. A negative *β*_2_ indicates that older age decreases the predicted *WRS*_65_(*CI*) [%], suggesting an age-related decline.

### 2.3. Data Analysis

We used the equation proposed by Hoppe et al. [11] to test whether this model can be used in a separate patient cohort. We calculated the *β* values of our cohort with a GLM to uncover differences between the cohorts. *β* values were calculated by linking the independent variables *WRS_max_*, age at implantation and *WRS*_65_(*HA*) to the dependent variable *WRS*_65_(*CI*). In line with the model proposed by Hoppe, *WRS*_65_(*CI*) was converted into a binary test result. For this, a table with a set of 20 rows (representing a Freiburg monosyllabic test list of 20 words) was generated for each patient. The preoperative variables remained the same across the rows, but the measured *WRS*_65_(*CI*) was converted to 1 and 0 in terms of the percentage of speech understanding.

Excel was used for data collection and calculation of values (e.g., predicted score, median absolute error, median improvement). Rstudio ([16], version 2024.12.1+563) was used to calculate the *β* values using the values for the variables *WRS_max_*, age at implantation and *WRS*_65_(*HA*) of our cohort, to test for the reliability of the prediction model. GraphPad Prism 10.3.0 (Graph Pad Software, LLC; San Diego, CA, USA) was used for statistical analysis and graph generation. Spearman’s rank correlation test was performed to assess the correlation between the predicted and the measured *WRS*_65_(*CI*).

## 3. Results

### 3.1. Characteristics of the Different Patient Subcohorts

In this study, 238 CI recipients were included. Table 2 presents a summary of the demographic and audiological characteristics of the study population. Patients were categorized into two groups: (1) those with a 4FPTA ≤ 80 dB HL and (2) the entire cohort, including all patients regardless of their 4FPTA. Within each group, data are presented separately for each cochlear implant manufacturer as well as for the total cohort encompassing all manufacturers.

### 3.2. Patients with a 4FPTA ≤ 80 dB HL

To externally validate the prediction model of Hoppe et al. [11] in an independent cohort, we included 24 patients of our cohort who met their inclusion criteria. These patients were German native speakers who received a CI from the manufacturer Cochlear and performed a 4FPTA lower or equal to 80 dB HL. In addition, 27 patients met the inclusion criteria except for the manufacturer; 25 patients received a CI from MED-EL and 2 from Advanced Bionics (AB).

The median *WRS*_65_(*CI*) for implanted patients meeting all inclusion criteria of Hoppe et al. [11] was 80%, with a median improvement of 17.5 percentage points (pp) compared with preoperative *WRS_max_*. With all 51 patients included, the respective values were median *WRS*_65_(*CI*) 75% and median improvement 15 pp.

**Figure 1:**
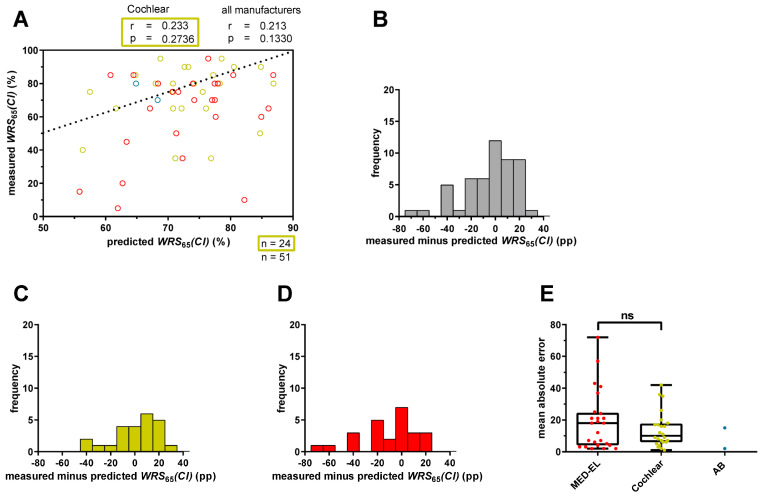
Prediction error for word recognition score at 65 dB with CI (*WRS*_65_(*CI*)) for patients with a four-frequency pure tone average (4FPTA) ≤ 80 dB HL. (**A**) The x-axis represents the predicted *WRS*_65_(*CI*), calculated with Equation (1), and the y-axis represents the measured *WRS*_65_(*CI*) after at least 12 months. The different colors represent different CI manufacturers (red: MED-EL; yellow: Cochlear; blue: Advanced Bionics (AB). The ideal fit between model predictions and measured values is indicated by the bisecting line. A deviation exceeding 20 percentage points indicates an inaccuracy in the prediction that has a relevant impact on patient counseling or expectation management. (**B**) The range of the prediction error for all patients (n = 51) was calculated by subtracting the predicted score from the measured score. Negative scores mean that the patients’ scores were below prediction and positive scores mean that the patients scored above prediction. (**C**) The range of the prediction error for only Cochlear™ recipients (n = 24) with a 4FPTA ≤ 80 dB HL. (**D**) The range of the prediction error for only MED-EL™ recipients (n = 25) with a 4FPTA ≤ 80 dB HL. (**E**) The mean absolute error for MED-EL™ (red), Cochlear™ (yellow) and AB™ (blue) recipients. ns—not significant.

Plotting the predicted *WRS*_65_(*CI*) against the measured scores showed a weak and statistically not significant correlation (r = 0.233; *p* = 0.2736) in the 24 Cochlear™ recipients (Figure 1A). Discrepancies between measured and predicted *WRS*_65_(*CI*) ranged from −42 pp to +26 pp, with a median absolute error (MAE) of 10 pp (Figure 1C,E). Negative values indicated worse speech recognition than predicted, while positive values indicated better performance (Figure 1C). In total, 75% of implantees had a *WRS*_65_(*CI*) better than −8 pp. Five patients performed at least 20 pp better or worse than predicted, and three of them performed at least 30 pp worse than predicted.

When we included 51 patients, including 27 who received cochlear implants from manufacturers other than Cochlear, the correlation between predicted and measured *WRS*_65_(*CI*) decreased and was still statistically not significant (r = 0.213; *p* = 0.1330; Figure 1A). The differences between measured and predicted *WRS*_65_(*CI*) increased, ranging from −72 pp to +26 pp (Figure 1B) with a median MAE of 11.6 pp. Only MED-EL™ recipients (n = 25) had a median MAE of 17.6 pp (Figure 1E). Relative to the predicted outcome, 16 patients (30.2%) deviated from the predicted score by at least 20 pp, and 9 of them (17%) by at least 30 pp.

When investigating the prediction accuracy depending on the CI manufacturer, we observed differences in the ranges of the prediction errors between MED-EL™ and Cochlear™ recipients (Figure 1C–E), with MED-EL™ recipients having a tendency towards higher prediction errors. However, these differences were not statistically significant (Figure 1E). Patients using a CI made by Advanced Bionics were excluded from statistical analyses due to the small number (n = 2) of patients included.

Thus, using the original inclusion criteria of Hoppe et al. [11], we found similar MAEs in our cohort. However, the inclusion of patients with CIs of different manufacturers suggested increased MAEs, although the differences were not statistically significant.

### 3.3. All Patients Regardless of 4FPTA

We then sought to assess the applicability of the predictive model to cochlear implant outcomes independent of tone audiometry. For this, inclusion criteria were extended to patients with a 4FPTA > 80 dB HL. Regardless of CI manufacturer, a total of 238 patients with bilateral profound hearing loss met the inclusion criteria.

Our cohort had a median *WRS*_65_(*CI*) of 75% and showed a median improvement of 40 pp. 

**Figure 2:**
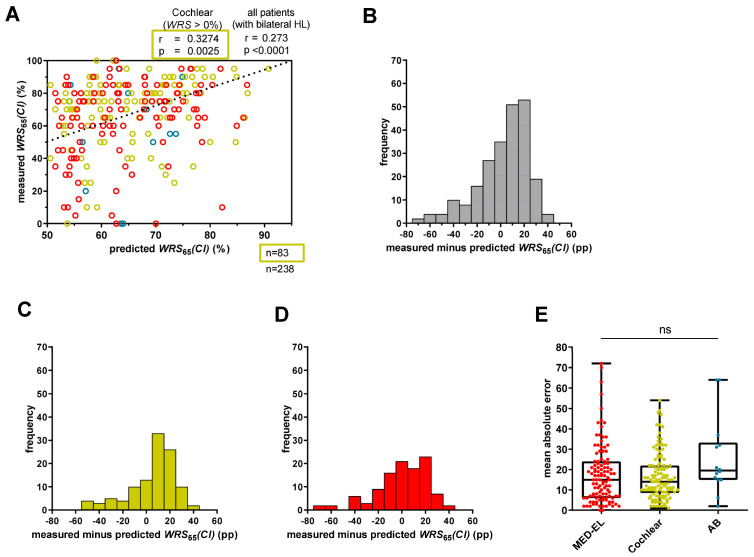
Prediction error for *WRS*_65_(*CI*) for all patients with bilateral hearing loss. (**A**) Predicted *WRS*_65_(*CI*), calculated with Equation (1), was plotted against measured *WRS*_65_(*CI*). The bisecting dotted line represents the ideal fit between model predictions and measured values. A deviation exceeding 20 percentage points indicates an inaccuracy in the prediction that has a relevant impact on patient counseling or expectation management. Red circles refer to MED-EL patients, yellow circles to Cochlear patients, and blue circles to AB patients. (**B**) The range of the difference between measured and predicted scores for all patients (n = 238). (**C**) The range of the prediction error for only Cochlear™ recipients (n = 113) with bilateral hearing loss. (**D**) The range of the prediction error for only MED-EL™ recipients (n = 111) with bilateral hearing loss. (**E**) The mean absolute error for MED-EL™ (red), Cochlear™ (yellow) and AB™ (blue) recipients. ns—not significant.

Figure 2A shows a weak correlation between measured and predicted scores (r = 0.273; *p* < 0.0001) for all patients, regardless of performance in *WRS_max_*. Differences between measured and predicted values had a large range from −72 pp to +41 pp (Figure 2B) resulting in a median MAE of 15 pp. Of note, approximately one third of the CI recipients deviated from the predicted score by more than 20 pp, either performing better (40 cases) or worse (38 cases) than predicted. Of the latter cases, 26 performed at least 30 pp worse than predicted.

We also examined whether the CI manufacturer influenced the prediction model. Although there were differences in the ranges between measured and predicted scores between MED-EL™ and Cochlear™ recipients (Figure 2C,D), as reflected by the median MAEs, statistical significance was not established (Figure 2E).

When only patients with a *WRS_max_* > 0% were included, Cochlear™ recipients (n = 83) showed a moderate correlation between measured and predicted *WRS*_65_(*CI*) (r = 0.3274; *p* = 0.0025), with a median MAE of 11.6 pp (Figure 2A). However, MED-EL™ recipients with a *WRS_max_* > 0% (n = 77) had a weak not statistically significant correlation (r = 0. 0.1960; *p* = 0.0876) but a comparable median MAE of 13.1 pp.

Overall, the model showed correlations but a limited overall accuracy for patients with a 4PTA > 80 dB HL when including all manufacturers. Regarding only Cochlear™ recipients, the correlation was moderate, but the median MAE was similar between MED-EL™ (14.9 pp) and Cochlear™ (14.5 pp) recipients. Similar results were shown for MED-EL™ and Cochlear™ recipients with a *WRS_max_* > 0%.

### 3.4. Comparison of Coefficients After Recomputing the GLM with Our Cohort

To evaluate which factors played a role in our cohort and the subgroups, we recomputed the parameters, including β-values, standard error, *t*-statistics and *p*-value, corresponding to the variables “*WRS_max_*”, “*age* at implantation” and “*WRS*_65_(*HA*)”.

First, the coefficients were computed for patients with a 4FPTA ≤ 80 dB HL, who received a CI from either Cochlear (Table 3, yellow) or MED-EL (Table 3, red) or all patients with a 4FPTA ≤ 80 dB HL (Table 3, apricot). For comparison, the values found by Hoppe et al. [11] are shown in white in Table 3. For Cochlear™ recipients with a 4FPTA ≤ 80 dB HL, all coefficients emerged as significant predictors, with *age* at implantation (*β*_2_) having the strongest effect. *WRS_max_* (*β*_1_) was positively correlated with *WRS*_65_(*CI*), while *β*_2_ and *WRS*_65_(*HA; β*_3_) were negatively correlated with *WRS*_65_(*CI*) (Table 3, yellow). In contrast, the coefficients for MED-EL™ recipients indicated that *WRS_max_* and *age* at implantation were significant predictors with low standard errors, while *WRS*_65_(*HA*) was not significant, as indicated by the *t*-statistic (Table 3, red highlights). Among all patients who met the inclusion criteria, regardless of manufacturer, *WRS_max_* and *age* at implantation showed significances with low variability. With the exception of *WRS*_65_(*HA*), the predictor variables proved to be important for the model, as the *t*-statistics showed high values. This was also supported by the *χ*^2^ statistics versus constant model. Coefficients were also calculated for the entire cohort, regardless of *WRS_max_* performance and 4FPTA (n = 238).

In the entire cohort and the different subgroups, *age* at implantation consistently had a negative effect of similar weight on postoperative speech understanding, with a coefficient *β*_2_ ranging from −0.029820 to −0.018061. *β*_1_, in contrast, consistently had a positive effect in all groups, with coefficients *β*_1_ ranging from 0.003240 to 0.008846. Depending on the subcohort, *WRS*_65_(*HA; β*_3_) coefficients showed controversial results, by having either a negative or positive effect on *WRS*_65_(*CI*), ranging from −0.010084 to 0.008952. A higher *β*_1_ was typically associated with a lower *β*_3_, indicating that interdependencies lead to positive effects on speech understanding with CI.

### 3.5. Exploring Maximum and Minimum Model Outputs

As our x-axes (Figure 1A and Figure 2A), as well as those in Hoppe et al. [11], were constrained to a range from 50% to 100%, we sought to assess the predictive limits of the model. To determine the maximum and minimum prediction score of this model using the estimates from Hoppe et al. [11], as well as estimates of this study, we used examples of two extremes. To calculate the best possible score, a patient with a *WRS_max_* and a *WRS*_65_(*HA*) each of 100% and an *age* at implantation of 0 years will result in a predicted *WRS*_65_(*CI*) of 93.3%. For the worst possible prediction, a patient with a *WRS_max_* and a *WRS*_65_(*HA*) each of 0% and an *age* at implantation of 100 years will result in a predicted *WRS*_65_(*CI*) of 47.5%. Using the recomputed coefficients for our cohort of Cochlear™ recipients with a 4FPTA ≤ 80 dB HL, the minimum score was 44.5% and the maximum score was 89%. Given these theoretical extreme values of the equation (100%, 0 years of age; 0%, 100 years of age), the model was not valid to predict low performers (below either 47.5% or 44.5%) before implantation. This limits its clinical value in the preoperative identification of patients at risk of poor cochlear implant outcomes.

## 4. Discussion

### 4.1. Prediction Model for Cochlear™ Recipients with a 4FPTA ≤ 80 dB HL

Applying the prediction model developed by Hoppe et al. [11] to our cohort provided valuable insights into its applicability and limitations. Our results shed light on the performance of the prediction model in different patient subgroups and revealed factors influencing its predictive accuracy.

Applying the prediction model to Cochlear™ recipients with a 4FPTA ≤ 80 dB HL demonstrated a weak statistically not significant correlation between measured and predicted *WRS*_65_(*CI*) (Figure 1A). Apart from that, deviations exceeding 20 pp were observed in a small subset of cases, which were considered to be clinically meaningful [17,18]. Comparable results were shown by Hoppe et al. [11]. Notably, our results revealed narrower ranges of differences between measured and predicted scores (Figure 1B), indicating that the model proposed by Hoppe et al. [11] results in a comparable fit accuracy for our cohort. A comparison between the regression summary of the model by Hoppe et al. [11] (Table 3, white) and the regression summary of our model for Cochlear™ recipients with a 4FPTA ≤ 80 dB HL (Table 3, yellow) revealed differences. In particular, our results showed a slightly smaller and significant predictor coefficient (*β*_1_), while the coefficients (*β*_2_ and *β*_3_) were higher and statistically significant. The larger standard errors in our cohort suggest greater variability in the underlying data compared with that reported by Hoppe et al. [11]. In addition, consistent trends in coefficient estimates confirmed the importance of the predictor variable *WRS_max_* in influencing speech understanding with CI, corroborating findings from previous studies [19,20].

### 4.2. Application of the Model to Patients with Profound Hearing Loss and Different Manufacturers

Extending the application of the model to our cohort of patients with profound hearing loss resulted in slightly worse predictions for a broader patient population. The correlation between measured and predicted scores remained weak (r = 0.273; *p* < 0.0001) (Figure 2A), with a notable proportion of scores showing substantial deviations and a median MAE of 15 pp (Figure 2E). Shafieibavani et al. [13] conducted a comparative analysis of different machine learning models to predict CI performance 12 months after surgery in 2489 recipients from three international clinics (Medizinische Hochschule Hannover [MHH]; Ear Science Institute Australia [ESIA]; Vanderbilt University Medical Center [VUMC]). They reported MAEs ranging from 20 to 22 pp within their cohorts [13]. A similar MAE was observed in a subset of patients with a *WRS_max_* = 0% [9]. The differences in MAEs between the Hoppe and Shafieibavani cohorts and our cohort could be attributed to several factors. First, 68 patients of our cohort with bilateral profound hearing loss (n = 238) had a *WRS_max_* of 0%, whereas the remaining participants had better performance (*WRS_max_* > 0%). Second, Shafieibavani and colleagues used different models with different predictor variables compared with our study and Hoppe’s study [9,13].

Another interesting finding was that Cochlear™ recipients with a 4FPTA ≤ 80 dB HL had a lower MAE (10 pp) (Figure 1E) compared with the overall cohort (15 pp) (Figure 2E), a trend consistent with the findings of Hoppe and colleagues [9,10]. In a recent study, they found that, while the original model was suitable for Cochlear™ recipients with a *WRS_max_* > 0%, those with a *WRS_max_* = 0% had an approximately doubled MAE. By including the duration of unaided hearing loss as an additional predictor variable, they were able to reduce the MAE from 23.7% to 17.2% [10]. Interestingly, they showed that modifying the original model to include all Cochlear™ recipients with a *WRS_max_* > 0% maintained a comparable MAE. Our results are consistent with this observation, as Cochlear™ recipients with a 4FPTA ≤ 80 dB HL (n = 24) had a median MAE of 10 pp (Figure 1E), while those with any 4FPTA and a *WRS_max_* > 0% (n = 83) had a median MAE of 11.6 pp. Thus, although the model can be applied to patients with a *WRS_max_* = 0%, it is associated with a slightly larger median absolute error [9]. These differences in MAEs might be attributed to differences in auditory nerve function. Patients with residual hearing typically have preserved auditory nerve function, whereas patients without residual hearing (*WRS_max_* = 0%) are characterized by reduced or, in rare cases, absent auditory nerve function [9,21].

The comparison of the summary of the model regression analyses from Hoppe et al. [11] (Table 3, white) and the Oldenburg cohort (Table 3, grey) showed that the coefficient *β*_1_ for our cohort was smaller but still statistically significant, indicating a smaller but substantial impact on the outcome [11]. The coefficients retained relevance, as evidenced by smaller standard errors, and reliability, as reflected by high absolute *t*-values.

Improvements in the predictive power of the published models seem to be indicated before applying them in clinical routine—a substantial proportion of patients exhibited notable differences between predicted and measured outcomes (Figure 2A,B). Consequently, only a portion of the variance in outcomes can be accounted for by the Hoppe et al. [11] model. As in our analyses, the Hoppe et al. [11] model also displayed variability in the data of the cohort [11]. Nevertheless, Hoppe and colleagues indicated that the model’s parameters are currently utilized in their clinical settings for quality assurance and preoperative counseling of CI candidates [9]. While the majority of patients benefit from CI, considerable variability in outcomes remains, with some patients unexpectedly failing to achieve any significant benefit. To illustrate the limitations of the model, we utilized hypothetical scenarios involving two patients, representing the best- and worst-case outcomes. We found that the model is not valid to predict CI outcomes below 47.5%. In terms of identifying unexpected poor performers, our data suggest that the prediction model has limitations. These limitations may be attributable to the model coefficients. Specifically, the intercept term (*β*_0_) is substantially larger compared with the coefficients of the predictor variables (*β*_1_–*β*_3_), suggesting that the influence of the predictors on the predicted CI outcome is relatively weak. Additionally, the presence of positive values, in combination with positive coefficients, contributes to higher predicted outcomes. Conversely, only *β*_2_, which represents a small negative value, exerts a reducing effect on the CI outcome. However, this effect is too small to reliably predict poor performers. Regarding clinical routine applications, improvements in the prediction models seem implicated.

### 4.3. Influence of CI Manufacturer

We examined whether the prediction model, originally trained with Cochlear™ recipients, also provides reasonable fits for different CI manufacturers—Cochlear, MED-EL and Advanced Bionics (AB). Due to the smaller number of recipients, AB was excluded from the analysis. A focused examination of Cochlear™ recipients revealed a weak not significant correlation between measured and predicted *WRS*_65_(*CI*) (r = 0.233; *p* = 0.2736). However, this correlation decreased when MED-EL™ and AB™ recipients were included (r = 0.213; *p* = 0.1330) (Figure 1A). Slight but not significant differences in MAEs were observed (Figure 1E and Figure 2E). As previously described, the MAE remained consistent between Cochlear™ recipients with a 4FPTA ≤ 80 dB HL (n = 24) and all Cochlear™ recipients with a *WRS_max_* > 0% (n = 83). Interestingly, for MED-EL™ recipients, the MAE decreased from 17.6 pp to 13.1 pp when extending the subgroup of 4FPTA ≤ 80 dB HL (n = 25) to MED-EL™ recipients with a *WRS_max_* > 0% (n = 77). Likely due to the limited sample size for each manufacturer subgroup with a 4FPTA ≤ 80 dB HL (Cochlear: n = 24; MED-EL: n = 25), the determination of the coefficients revealed slight variations (Table 3). Specifically, for Cochlear™ recipients, all coefficients—*WRS_max_*_,_ *age* at implantation and *WRS*_65_(*HA*)—emerged as important predictors. For MED-EL™ recipients, both *WRS_max_* and *age* at implantation were significant predictors for *WRS*_65_(*CI*), the latter having the strongest effect. Possible explanations for the small differences between manufacturers could be attributed to various factors, including individual cochlear morphology, surgical technique, electrode placement, insertion depth, number of active electrodes, microphone sensitivity and compression implementation [5,14,22,23]. However, the magnitude of the differences between the manufacturers was minimal, leading to the conclusion that the model is more adept at predicting outcomes for Cochlear™ recipients with functional residual hearing (*WRS_max_* > 0%) but is also applicable to MED-EL™ recipients under similar conditions, albeit with a higher deviation from prediction. These findings are, among other factors, limited by the relatively small patient cohorts, indicating that national and/or international collaborative efforts would increase the power of predictive models, particularly in the subgroup of AB™ recipients. To increase the statistical power and generalizability of predictive models, larger, multicenter datasets—whether national or international in scope—are needed through collaborative research efforts. 

### 4.4. Generalizability of the Prediction Model

The accuracy of this predictive model depends on several factors, such as functional residual hearing and, to some extent, the manufacturer. Our study, along with Hoppe et al. [11], has shown that the best fit of the model is for patients with a 4FPTA ≤ 80 dB HL who have received an implant from the manufacturer Cochlear. Extending the model to Cochlear™ recipients with any 4FPTA but at least some residual speech understanding (*WRS_max_* > 0%) yielded only slightly worse results. Also, when applying the model to the entire cohort, a considerable number of patients deviated from the predictions supported by the weak correlation between measured and predicted scores (r = 0.273; *p* < 0.0001) (Figure 2). Calculation of the *β* values for the Oldenburg cohort showed lower estimates compared with those of Hoppe et al. [11]. The differences in parameters and goodness of fit also suggest that caution should be exercised when applying the model to another cohort (Table 3). However, for both cohorts—ours and the Hoppe cohort—the predictors (*β*_1_, *β*_2_, *β*_3_) played a role in capturing additional variability and providing a deeper explanation for differences in speech recognition scores beyond this baseline. The predictive factors *β*_1_, *β*_2_, *β*_3_ all significantly increased the explanatory power of the model, ensuring a more accurate and comprehensive understanding of the factors influencing speech recognition outcomes.

Models with backward selection showed that not only preoperative measurements and demographic variables are relevant factors influencing postoperative speech perception abilities [6] but factors such as some etiologies of hearing loss [3] and perioperative circumstances (electrode insertion depth, problems encountered during surgery) have been shown to have an impact [5,14,22,23]. This model may need to be adjusted to include additional predictor variables. Additional variables, such as postoperative categorical loudness scaling and hearing loss for Freiburg numbers, obtained good correlations [8]. Duration of unaided hearing impairment as another predictor variable was also shown to lead to a decrease in the MAE compared with the original model [10]. Subsequent studies should focus on externally validating this extended model [10], including the duration of unaided hearing impairment, and testing its applicability on a cohort with a wider variety of characteristics to ensure its robustness and generalizability in diverse clinical settings. A disadvantage, however, is that the assessment of the duration of the untreated hearing impairment depends on the quality of the patient’s report and is therefore subject to greater uncertainty.

## 5. Conclusions

Our study provides significant insights into the applicability and limitations of the prediction model proposed by Hoppe et al. [11]. We found comparable results for Cochlear™ recipients, particularly those with a PTA4 ≤ 80 dB HL and, independently of tone audiometry, for recipients with a *WRS_max_* > 0%. Extending the model to additional manufacturers or patients with a *WRS_max_* ≥ 0%, independent of tone audiometry, resulted in similar predictions, though with slightly higher prediction errors.

However, the model’s limitations are evident, as it cannot predict scores below 47.5%, rendering it unsuitable for identifying poor performers. This is an important limitation, as identifying patients at risk of poor performance is a key function of a prediction model. Despite these challenges, the model has potential for specific patient subgroups, though further work is needed to enhance its robustness across broader populations and different CI manufacturers.

Future research should aim to integrate these established predictors with emerging findings from neuroimaging, genetic studies and cognitive evaluations. Developing more accurate predictive models will require a collaborative multidisciplinary approach, drawing on expertise from audiology, otology, neurology and data analytics. Such models will enable improved patient selection, personalized counseling and more effective rehabilitation strategies, ultimately enhancing cochlear implant outcomes. Additionally, further research focused on external validation is essential to refine the predictive accuracy and generalizability of the model for clinical application.

## Figures and Tables

**Table 1:** Parameters of the generalized linear model (GLM) created by Hoppe et al. [11].

	Estimate	Std. Error	*t*-Statistics	*p*-Value	[β]
*β*_0_	0.84	0.18	4.59	4 × 10^−6^	
*β*_1_	0.012	0.0015	8.07	7 × 10^−16^	1/%
*β*_2_	−0.0094	0.0025	−3.72	2 × 10^−4^	1/year
*β*_3_	0.0059	0.0026	2.30	2 × 10^−2^	1/%

A total of 5120 observations and 5116 error degrees of freedom. χ^2^ statistic versus constant model: 157, *p*-value = 9 × 10^−34^.

**Table 2:** Demographic and audiological characteristics of the patient cohort.

	Number of Patients (n)	Age at Implantation (Years) (Median [Min–Max])	4FPTA (dB HL) (Median [Min–Max])	*WRS_max_* (%) (Median [Min–Max])	*WRS*_65_(*HA*) (%) (Median [Min–Max])	*WRS*_65_(*CI*) (%) (Median [Min–Max])
Patients with a 4FPTA ≤ 80 dB HL						
Cochlear	24	66 [41–79]	73.13 [55–80]	52.50 [10–100]	25 [0–65]	80 [35–95]
MED-EL	25	66 [47–82]	73.75 [63.75–80]	55 [5–95]	25 [0–75]	70 [5–95]
AB	2	70 [68–72]	78.13 [77.5–78.75]	35 [35–35]	15 [5–25]	75 [70–80]
all	51	66 [41–82]	73.75 [55–80]	55 [5–100]	25 [0–75]	75 [5–95]
All patients regardless of 4FPTA						
Cochlear	113	64 [21–87]	93.75 [55–120]	20 [0–100]	5 [0–95]	75 [0–100]
MED-EL	111	69 [36–84]	91.25 [63.75–120]	25 [0–95]	10 [0–75]	70 [0–100]
AB	14	70 [49–78]	90.63 [77.5–117.5]	20 [0–65]	7.5 [0–40]	62.5 [0–95]
all	238	67 [21–87]	92.5 [55–120]	22.5 [0–100]	5 [0–95]	75 [0–100]

**Table 3:** Parameters of the GLM different groups of patients of the Oldenburg cohort.

	Patient Group		Estimate	Std. Error	*t* Statistics	*p*-Value	[β]
Parameters from Hoppe et al. [11]	Cochlear (n = 128)	*β*_0_	0.84	0.18	4.59	4 × 10^−6^	
		*β*_1_	0.012	0.0015	8.07	7 × 10^−16^	1/%
		*β*_2_	−0.0094	0.0025	−3.72	2 × 10^−4^	1/year
		*β*_3_	0.0059	0.0026	2.30	2 × 10^−2^	1/%
		5120 observations; error degrees of freedom: 5116; *χ*^2^ statistics versus constant model: 157; *p*-value = 9 × 10^−34^					
4FPTA ≤ 80 dB HL	Cochlear (n = 24)	*β*_0_	2.850196	0.504685	5.647	1.63 × 10^−8^ ***	
		*β*_1_	0.006701	0.003365	1.992	0.0464 *	1/%
		*β*_2_	−0.029820	0.006562	−4.544	5.52 × 10^−6^ ***	1/year
		*β*_3_	−0.010084	0.004138	−2.437	0.0148 *	1/%
		960 observations; error degrees of freedom: 956; *χ*^2^ statistics versus constant model: 26.04117; *p*-value = 9.349953 × 10^−6^					
	MED-EL (n = 25)	*β*_0_	1.111776	0.514790	2.160	0.03080 *	
		*β*_1_	0.008846	0.004062	2.178	0.02943 *	1/%
		*β*_2_	−0.018061	0.006831	−2.644	0.00819 **	1/year
		*β*_3_	0.003403	0.004851	0.702	0.48290	1/%
		1000 observations; error degrees of freedom: 996; *χ*^2^ statistics versus constant model: 31.068; *p*-value = 8.224792 × 10^−7^					
	All manufacturers (n = 51)	*β*_0_	1.985175	0.344830	5.757	8.56 × 10^−9^ ***	
		*β*_1_	0.008836	0.002423	3.646	0.000266 ***	1/%
		*β*_2_	−0.024309	0.004590	−5.297	1.18 × 10^−7^ ***	1/year
		*β*_3_	−0.004243	0.002971	−1.428	0.153241	1/%
		2040 observations; error degrees of freedom: 2036; *χ*^2^ statistics versus constant model: 50.07659; *p*-value = 7.694683 × 10^−11^					
Entire cohort	(n = 238)	*β*_0_	1.284117	0.132794	9.670	<2 × 10^−16^ ***	
		*β*_1_	0.003240	0.001021	3.174	0.0015 **	1/%
		*β*_2_	−0.012392	0.001889	−6.561	5.36 × 10^−11^ ***	1/year
		*β*_3_	0.008952	0.001643	5.450	5.04 × 10^−8^ ***	1/%
		9520 observations; error degrees of freedom: 9516; *χ*^2^ statistics versus constant model: 171.5774; *p*-value = 5.809114 × 10^−37^					

Significance levels are indicated as follows: *p* < 0.05 (*), *p* < 0.01 (**), and *p* < 0.001 (***).

## Data Availability

The data presented in this study are available on request from the corresponding author. The data are not publicly available due to privacy restrictions.
